# DNA-binding proteins studied by mechanical manipulation and AFM imaging of single DNA molecules

**DOI:** 10.52601/bpr.2022.220015

**Published:** 2022-08-31

**Authors:** Xiaodan Zhao, Xuyao Priscilla Liu, Jie Yan

**Affiliations:** 1 Department of Physics, National University of Singapore, Singapore 117542, Singapore; 2 Mechanobiology Institute, National University of Singapore, Singapore 117411, Singapore; 3 Centre for Bioimaging Sciences, National University of Singapore, Singapore 117546, Singapore

**Keywords:** AFM imaging, Single-molecule manipulation, Magnetic tweezers, Optical tweezers, DNA–protein interactions, DNA supercoiling, DNA distortion

## Abstract

The functions of DNA-binding proteins are dependent on protein-induced DNA distortion, the binding preference to special sequences, DNA secondary structures, the binding kinetics and the binding affinity. Recent rapid progress in single-molecule imaging and mechanical manipulation technologies have made it possible to directly probe the DNA binding by proteins, footprint the positions of the bound proteins on DNA, quantify the kinetics and the affinity of protein–DNA interactions, and study the interplay of protein binding with DNA conformation and DNA topology. Here, we review the applications of an integrated approach where the single-DNA imaging using atomic force microscopy and the mechanical manipulation of single DNA molecules are combined to study the DNA–protein interactions. We also provide our views on how these findings yield new insights into understanding the roles of several essential DNA architectural proteins.

## INTRODUCTION

DNA-binding proteins play a major role in the physical organization of genomic structures, regulations of gene transcription, DNA replication, and DNA-damage repair (Bannister and Kouzarides 2011; Dame 2005; Luijsterburg* et al.* 2008). The biological functions of these DNA-binding proteins rely on the basic DNA–protein recognition and interactions. These interactions may cause different types of DNA distortion on the binding sites and have different binding preferences to DNA sequences, DNA topology, and the secondary DNA structures such as double-stranded DNA (dsDNA), G-quadruplexes, and Holliday junction.

Traditional biochemical assays of DNA–protein interactions are often performed in bulk, such as electrophoretic mobility shift assays (Fried and Crothers 1981), footprinting (Galas and Schmitz 1978), fluorescence anisotropy (Jameson and Ross 2010; Royer and Scarlata 2008; Weber 1952), isothermal titration calorimetry, stop-flow fluorimetry, surface plasmonic resonance (Fagerstam* et al.* 1992), protein binding microarrays (Berger* et al.* 2006) and so on. Such measurements typically provide static ensemble average information of DNA–protein interactions and often do not inform us of the DNA distortion induced by protein binding. As the kinetics of protein binding to DNA and the conformational changes of DNA induced by protein binding are often closely related to the functions of the proteins, there is a gap between the knowledge derived from the biochemical bulk assays and the physical mechanisms underlying the functions of DNA-binding proteins.

The recent rapid development of single-molecule methods has made it possible to probe DNA–protein interactions at a single DNA level. Single-DNA imaging techniques can visualize the conformation of individual DNA molecules and proteins bound to the DNA. Single-DNA mechanical manipulation techniques can quantify the dynamic DNA–protein interactions at a single DNA or a single DNA-binding site level. While each has been extensively applied in the studies of DNA–protein interactions, the integration of the two different types of single-molecule methods has been shown more powerful in unveiling the DNA-binding properties of various proteins.

In this article, we review the applications of combining the single-DNA imaging using atomic force microscopy (AFM) with the single-DNA mechanical manipulation in the studies of DNA–protein interactions. We first provide an overview of AFM imaging and the single-molecule manipulation technologies, which is followed by a review of the quantification methods using those technologies in DNA–protein interactions at a single DNA level. We then demonstrate the examples of several recently published studies on the DNA-binding architectural proteins.

## AFM IMAGING AND MECHANICAL MANIPULATION OF DNA MOLECULES

### AFM imaging of DNA–protein complexes

**Figure 1:**
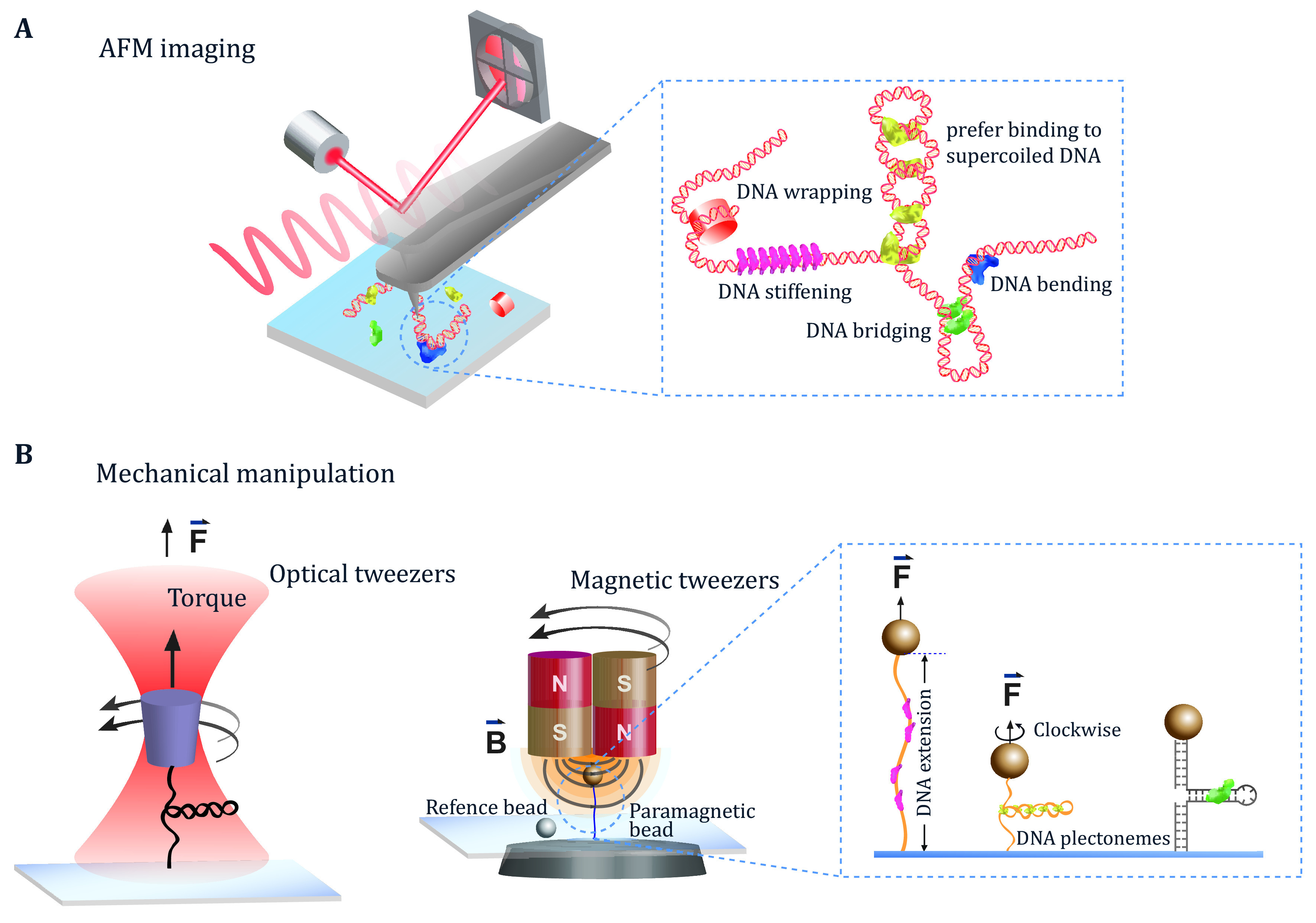
Schematics of AFM imaging (**A**) and the mechanical manipulation of single DNA molecules using optical tweezers and magnetic tweezers (**B**). Zoom-ins in Panels A and B show the representative conformations of DNA–protein complexes

AFM uses a sharp tip attached to a cantilever to scan a surface and provide the topography information of individual DNA molecules and DNA–protein complexes. Tapping mode and peak force mode are commonly applied to image biological molecules in dry or liquid conditions, which minimizes the sample disturbance and thus shows a nanometer lateral resolution (Binnig* et al.* 1986; Gerber and Lang 2006; Hansma* et al.* 1995, 2000; Lal and John 1994) (Fig. 1A). In addition, the tip-surface interaction is sensitive to the tip-surface distance; hence, AFM can also quantify the height of the molecules relative to the surface at a sub-nanometer vertical resolution (Dufrene* et al.* 2017; Goodman and Garcia 1991; Hinterdorfer and Dufrene 2006). Owing to the high lateral and vertical resolutions, AFM can identify proteins bound on the DNA molecules, quantify the local DNA deformation at the protein-bound sites, and probe the physical organization of a large DNA by the bound proteins. While being powerful in providing the conformational information of the DNA–protein complexes, there are several limitations of AFM imaging in the studies of DNA-binding proteins. The limited scanning rate of most of the commercially available AFM instruments makes typical AFM imaging only provide the static conformational information of DNA–protein complexes. Further, there is a concern that the strong DNA–surface interaction needed for its two-dimensional confinement would significantly influence the DNA–protein interactions.

### Single-DNA mechanical manipulation studies of protein binding to a DNA molecule under the mechanical constraints

Single-molecule mechanical manipulation refers to the ability of applying the mechanical constraints to a biomolecule and measure its responses to the applied mechanical constraints (Neuman and Nagy 2008). Figure 1B illustrates the optical tweezers and the magnetic tweezers, which are two commonly applied single-molecule manipulation technologies for the DNA–protein interactions. The typical mechanical constraints that can be applied to DNA molecules with their corresponding mechanical responses are summarized in Box 1.

**Table:** 

**Box 1| Mechanical constraints**
Commonly applied mechanical constraints include the constraint of: (1) an external force F (F-constraint), (2) the position of an external Hookean spring R attached to one end of a tethered DNA molecule (R-constraint), (3) a fixed linking number (Lk-constraint), and (4) a constant torque applied to a DNA molecule (T-constraint). More than one mechanical constraint can be applied simultaneously to the DNA molecule in an experiment. The typical measurable mechanical responses depend on the types of the mechanical constraints applied. Under the F- and R-constraints, the measurables are the extension of DNA and the tension in the DNA molecules. Under the Lk- and T-constraints, the measurables are the extension, torque, and the linking number of a double-stranded DNA molecule (dsDNA). Due to the convenience of experimental measurement, the DNA extension is the mostly used measurable responding to the above mentioned mechanical constraints.

**Figure 2:**
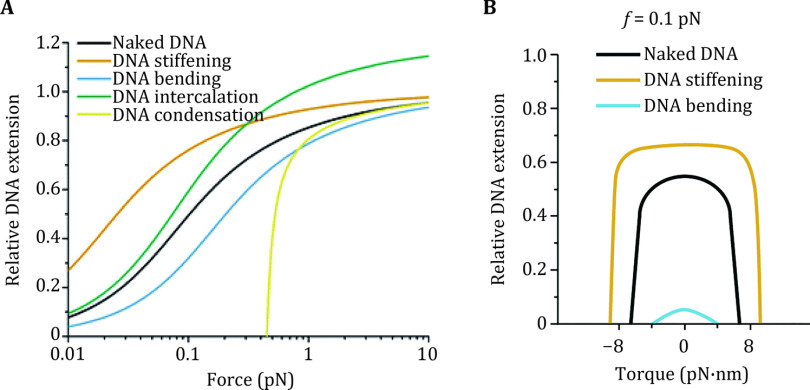
Extension changes in DNA molecules under the constraints of force (**A**) and torque (**B**), in response to different DNA distorting architectural proteins

The DNA–protein interactions can be probed based on the shifts in the mechanical responses of single DNA molecules upon protein binding, when a mechanical constraint is applied at a high spatial resolution (nanometer for the extension, a few degrees for the rotation, pN for the tension, and $ \mathrm{p}\mathrm{N}\cdot \mathrm{n}\mathrm{m} $ for the torque) and a high temporal resolution (hundreds to thousands of Hz). Therefore, single-DNA mechanical manipulation can probe the dynamic aspects of the DNA–protein interactions. Yet, there are limitations in this approach. The shifts in the mechanical responses are required to be sufficiently large for the detection. As DNA distortion occurring at a single binding site is typically small, this approach is often applied to probe the non-specific protein binding to a large DNA molecule which allows DNA binding by many proteins. Figure 2 illustrates the shifts in the extension of a dsDNA under the constraints of a force (Fig. 2A) and a torque (Fig. 2B), with proteins that cause different types of DNA distortions. The profile of the extension of DNA as a function of linking number density is expected to be similar to that of Fig. 2B. As mechanical constraints are applied to the DNA molecule, both the DNA conformation and DNA–protein interactions are determined by the mechanical constraints. Compared to the bulk measurements where the DNA molecules are not subject to the mechanical constraints, the single-DNA mechanical manipulation under different mechanical constraints yields results that require extrapolation with models to be comparable (Wang* et al.* 2019).

### Single-DNA mechanical manipulation studies of protein binding to a specific DNA site in the absence of the mechanical constraints

**Figure 3:**
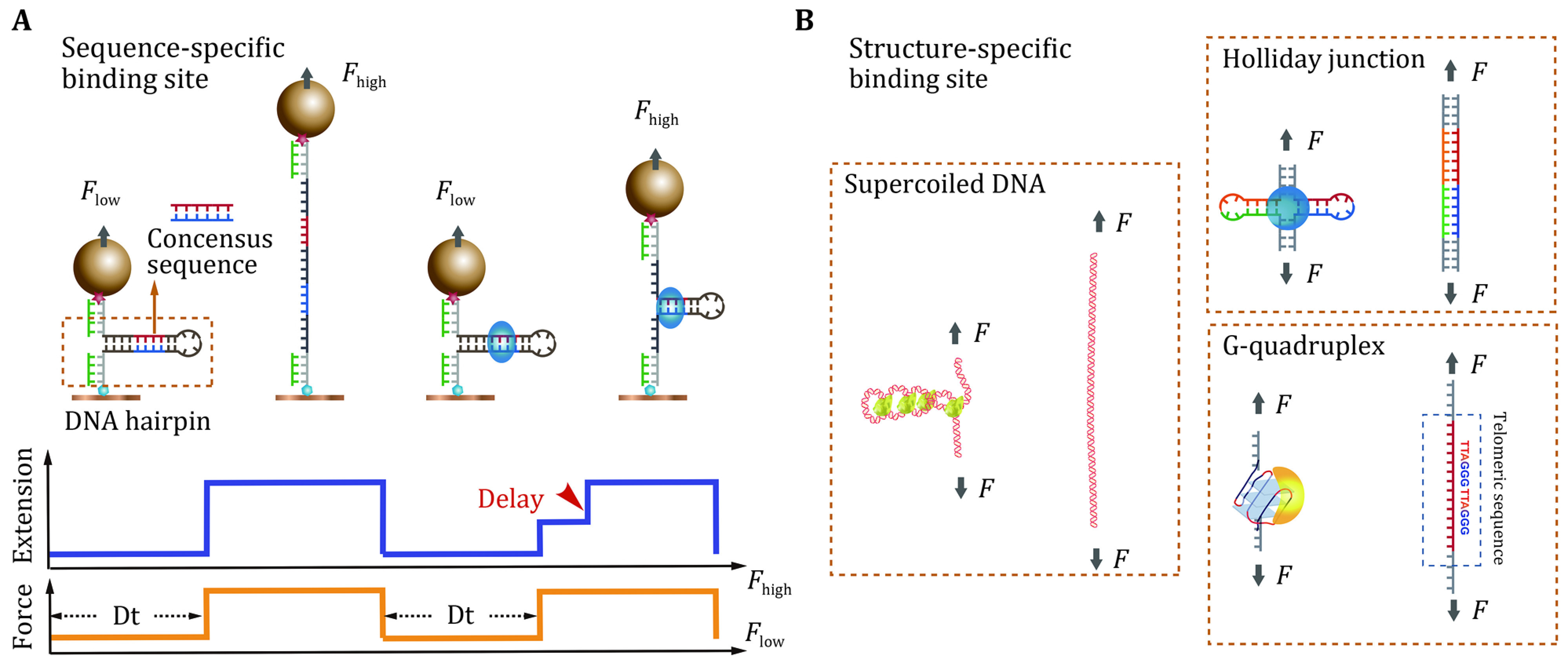
Schematics of detecting the site-specific protein binding via the single-DNA mechanical manipulation, where the binding site is excluded from the force transmission pathway. **A** The detection of binding using a DNA hairpin detector is based on the delayed unzipping upon an increased hairpin-destabilizing force. **B** The same approach can be extended to quantify the protein binding to specific DNA conformations and structures such as supercoiled DNA plectonemes, Holliday junctions, and G-quadruplexes

Site-specific protein binding to DNA can also be studied under a force-free condition using the single-DNA mechanical manipulation approach. High-affinity protein binding to a specific sequence or a specific DNA structural motif may stabilize the corresponding binding site, which in turn decreases the rate of the structural changes of that site under a destabilizing force applied to it. This shift in the kinetics of DNA structural changes can be utilized to quantify the protein binding to a specific DNA site. Thus, a single-DNA construct containing a specific protein binding site which is excluded from the force-transmission pathway is designed to quantify the interaction between the protein and the specific DNA site in the absence of the mechanical constraints. For example, under a sufficiently small force applied to a dsDNA hairpin spanned between two handles, the hairpin is stable and excluded from the force transmission pathway (Fig. 3A). Hence, the applied force does not affect the protein binding to the DNA site in the hairpin. Then, the hairpin is unzipped under a force greater than a threshold value that destabilizes the DNA hairpin. The rate of unzipping will be significantly decreased if the DNA site is stably bound by a protein. In this way, the protein-binding induced delay in the unzipping of the DNA hairpin can be used to determine the presence of a bound protein in the hairpin. This approach has been utilized for footprinting the DNA binding sites (Gulvady* et al.* 2018; Koch* et al.* 2002; Liang* et al.* 2021) and quantifying the binding affinities of DNA-binding proteins (Gulvady* et al.* 2018; Manosas* et al.* 2017; Zhao* et al.* 2017).

Importantly, the protein binding kinetics can also be obtained via unzipping a DNA hairpin through force-jump cycles (Fig. 3A). Varying the time duration ($ \Delta t) $ during which the proteins freely dissociate from/associate to the DNA hairpin, the probability of the hairpin bound by a protein, $ p\left(\Delta t\right) $, can thus be quantified as the fraction of the force-jump cycles where the binding occurs. At a given protein concentration ($ c $), the association rate ($ {k}_{\mathrm{o}\mathrm{n}} $) and the dissociation rate ($ {k}_{\mathrm{o}\mathrm{f}\mathrm{f}} $) can be obtained by fitting the experimentally obtained $ p\left(\Delta t\right) $ to a theoretical binding curve,

$ \;p\left(\Delta t\right)=\frac{c{k}_{\rm{o}\rm{n}}}{c{k}_{\rm{o}\rm{n}} + {k}_{\rm{o}\rm{f}\rm{f}}}\left(1-{e}^{-\left(c{k}_{\rm{o}\rm{n}} + {k}_{\rm{o}\rm{f}\rm{f}}\right)\Delta t}\right). $

The dissociation constant is thus obtained as $ {K}_{d}={k}_{\mathrm{o}\mathrm{f}\mathrm{f}}/{k}_{\mathrm{o}\mathrm{n}} $. A similar force-jump assay can be extended to quantitatively analyze the protein binding to other specific DNA sites such as the supercoiled DNA, G-quadruplexes, and the Holliday junctions, as illustrated in Fig. 3B.

Together, the integration of AFM imaging and the single-DNA mechanical manipulation technologies on DNA–protein complexes can provide a comprehensive understanding of the conformation, the modes, the kinetics and the binding affinity of proteins in DNA–protein interactions. The following recent studies using this integrated approach on several DNA architectural proteins demonstrate that they play critical roles not only in genome organization when abundantly present, but also in highly specific biological processes when targeting a specific sequence, a topological state, or a structural motif.

## APPLICATIONS ON DNA–PROTEIN INTERACTIONS

### Non-specific DNA-binding by histone octamers and nucleoid associated proteins

The basic units of the genome organization in mammalian cells are nucleosomes, each consisting of a histone octamer and a short stretch (~147 bp) of DNA wrapping around the octameric core by ~1.7 turns with a left-handed chirality (Bednar* et al.* 1998; Davey* et al.* 2002; Kornberg 1977; Luger* et al.* 1997). Nucleosomes are separated by linker DNAs of 15–50 bp and are associated with the linker histone H1. The linear arrays of nucleosomes are further coiled into thicker, higher-order chromatin fibers depending on the solution conditions, other chromatin-associated proteins, and the post-transcriptional histone modifications (Blank and Becker 1996; Holde 1989; Widom 1989; Woodcock and Dimitrov 2001).

**Figure 4:**
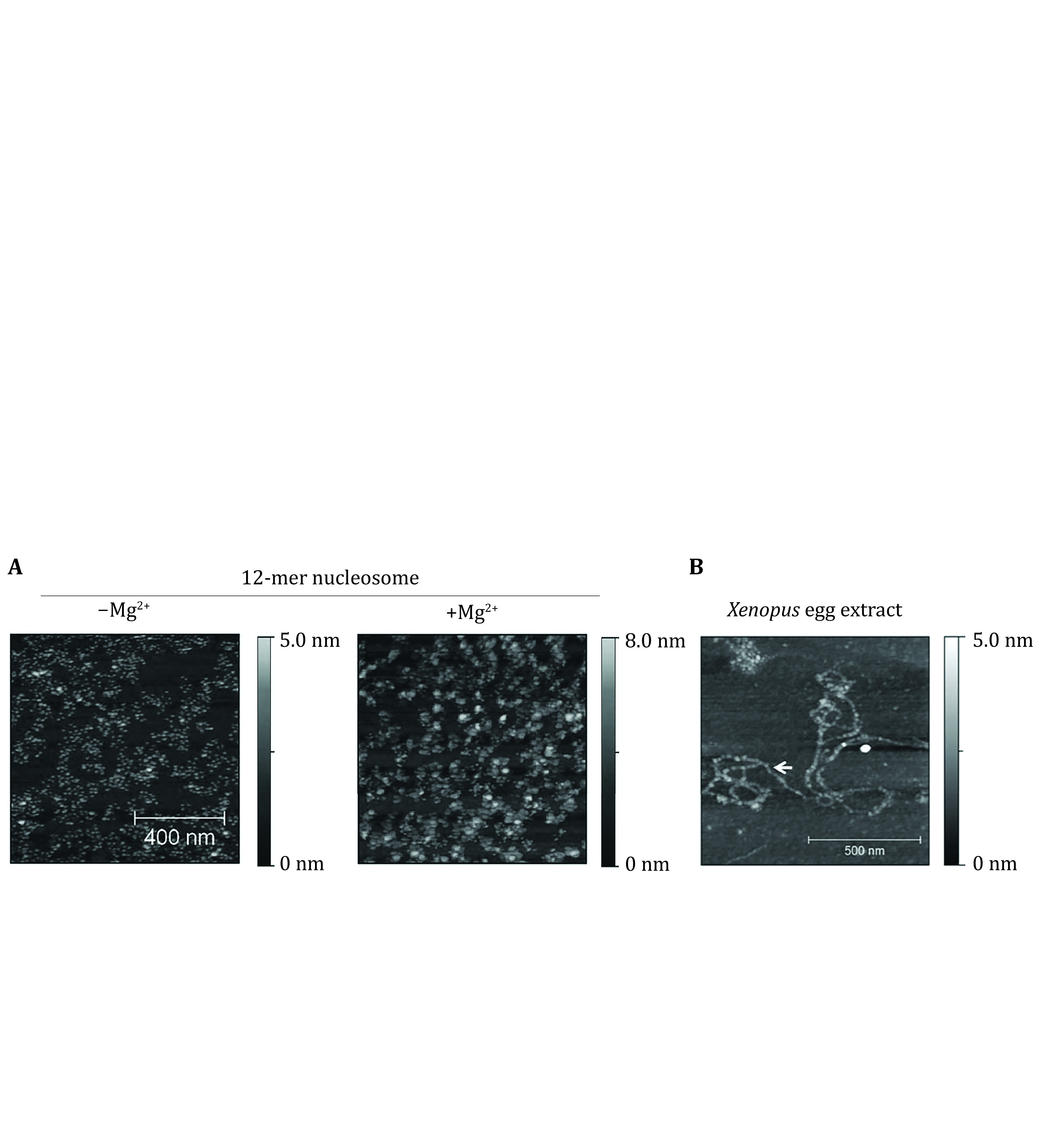
**A** AFM images of 12-mer nucleosomes assembled in the presence and absence of Mg^2+ ^ (unpublished). **B** An AFM image of chromatin assembled on 49-kbp $ \lambda $-phage DNA in *Xenopus* egg extract (Fu* et al.* 2011)

Reconstituted mono-nucleosomes, linear arrays of nucleosomes, and the higher-order chromatin structures have been extensively studied using AFM imaging (Krzemien* et al.* 2017; Poirier* et al.* 2008; Wang* et al.* 2002) and the single-DNA manipulation technologies (Brent Brower-Toland 2004; Hall* et al.* 2009; Kaczmarczyk* et al.* 2018, 2020; Kruithof* et al.* 2009; Li* et al.* 2016). Figure 4A shows the AFM imaging of 12-mer nucleosome arrays formed on DNA with 12 repeats of 601 sequences in the absence or presence of 1 mmol/L $ \mathrm{M}{\mathrm{g}}^{2 + } $ (unpublished). The AFM imaging also revealed similar beads-on-a-string chromatin structures assembled upon mixing DNA molecules with *Xenopus* egg extract (Fu* et al.* 2011).

The DNA wrapping around a histone octamer is mediated by strong attractive electrostatic interactions in a largely sequence independent manner. The dynamics, stability and the chirality of the nucleosomes have been investigated by the single-DNA mechanical manipulations (Brent Brower-Toland 2004; Germond* et al.* 1975; Hall* et al.* 2009; Kaczmarczyk* et al.* 2018, 2020; Kruithof* et al.* 2009; Kruithof and van Noort 2009; Li* et al.* 2016; Mihardja* et al.* 2006; Ngo* et al.* 2015; Norton* et al.* 1989; Simpson* et al.* 1985; Vlijm* et al.* 2012, 2015; Yan* et al.* 2007). Pre-assembled nucleosomes were reported to unfold with two steps at different forces. At forces slightly above 3.5 pN, the outer turn of DNA unwraps from the histone octamer, and the process is reversible when force is reduced. DNA unwrapping is irreversible when forces are above 15 pN, during which not only the inner turn of DNA is unwrapped but also leads to the destabilization of the histone octamer and its dissociation from DNA (Kruithof and van Noort 2009; Mihardja* et al.* 2006; Ngo* et al.* 2015; Yan* et al.* 2007). In addition, the DNA–histone interactions along the DNA sequence can be obtained by mechanically unzipping a DNA hairpin containing a single nucleosome on it. The study using nucleosomes formed on the high-affinity 601 DNA sequence revealed a distinct periodicity of ~5 bp in DNA–histone interactions, with the strongest at the dyad and the other two at ~± 40 bp from the dyad. Such studies provide us the information on the critical DNA–histone interactions that give rise to the stability of nucleosomes (Hall* et al.* 2009).

The left-handed chiral wrapping of DNA in nucleosomes is manifested in single-DNA supercoiling studies. At sub-pN forces, the formation of nucleosomes was measured at different linking number densities, $ \sigma =\dfrac{\Delta Lk}{L{k}_{0}} $, where $ \Delta Lk=Lk-L{k}_{0} $ is the change of the linking number ($ Lk $) from the relaxed state of DNA $\left( L{k}_{0}=\dfrac{number \; of \; basepairs}{10.4} \right)$. The resulting $ \sigma $-extension curve of DNA has a concave bell-like profile with a peak at $ \sigma =0 $ for the linking number of unconstrained DNA molecules, and $ \sigma  < 0 $ for the nucleosome formation on DNA due to its left-handed chirality. Based on the shift of $ \sigma $ from 0 and the number of nucleosomes formed on a DNA molecule, on average, each nucleosome contributes to a change of the linking number by roughly –1.2 and a 56 $ \pm $ 3 nm decrease in length per negative unit change (Vlijm* et al.* 2012), which is consistent with that determined in bulk supercoiling assays ($ \Delta Lk=-1.0 $) (Germond* et al.* 1975; Norton* et al.* 1989; Simpson* et al.* 1985). Applying a near-zero torque to the DNA and monitoring the number of turns in DNA rotation using freely orbiting magnetic tweezers (Lipfert* et al.* 2011), the linking number change per nucleosome formation was directly measured as –1.2 $ \pm $0.3 (Vlijm* et al.* 2015).

The integrated AFM and single-DNA manipulation approach has also been applied to investigate several abundantly expressed nucleoid-associated proteins (NAPs) that are responsible for genome packing in bacterial cells (Lim* et al.* 2012a, b; Liu* et al.* 2010; Winardhi* et al.* 2012, 201) and play an important role in globally regulating the bacterial gene expression (Browning* et al.* 2010; Dame 2005; Dillon and Dorman 2010; Luijsterburg* et al.* 2006; Winardhi* et al.* 2015). Due to the high concentrations, NAPs bind DNA in a largely sequence-independent manner. Some of the NAPs have a stronger binding affinity to specific DNA sequences in the promoters of certain genes and hence have significant impacts on the expression level of the corresponding genes (Bouffartigues* et al.* 2007; Hales* et al.* 1994; Lang* et al.* 2007; Prieto* et al.* 2012).

#### H-NS protein

H-NS family proteins play essential roles in shaping the bacterial nucleoid (Dame* et al.* 2000; Liu* et al.* 2010; Spurio* et al.* 1992). The proteins typically consist of a C-terminal DNA-binding domain, which preferentially binds the specific sequence motifs and a coiled-coil N-terminal domain that mediates the oligomerization, leading to higher-order homomeric complexes (Arold* et al.* 2010; Renault* et al.* 2013). The N-terminal oligomerization domain and the C-terminal DNA-binding domain are joined via an unstructured flexible linker with several positively charged residues. Besides providing flexibility to H-NS, the linker could also participate in DNA binding via a strong non-specific electrostatic interaction between the positively charged residues and the negatively charged sugar-phosphate backbone of the DNA molecule (Gao* et al.* 2017; Gulvady* et al.* 2018).

**Figure 5:**
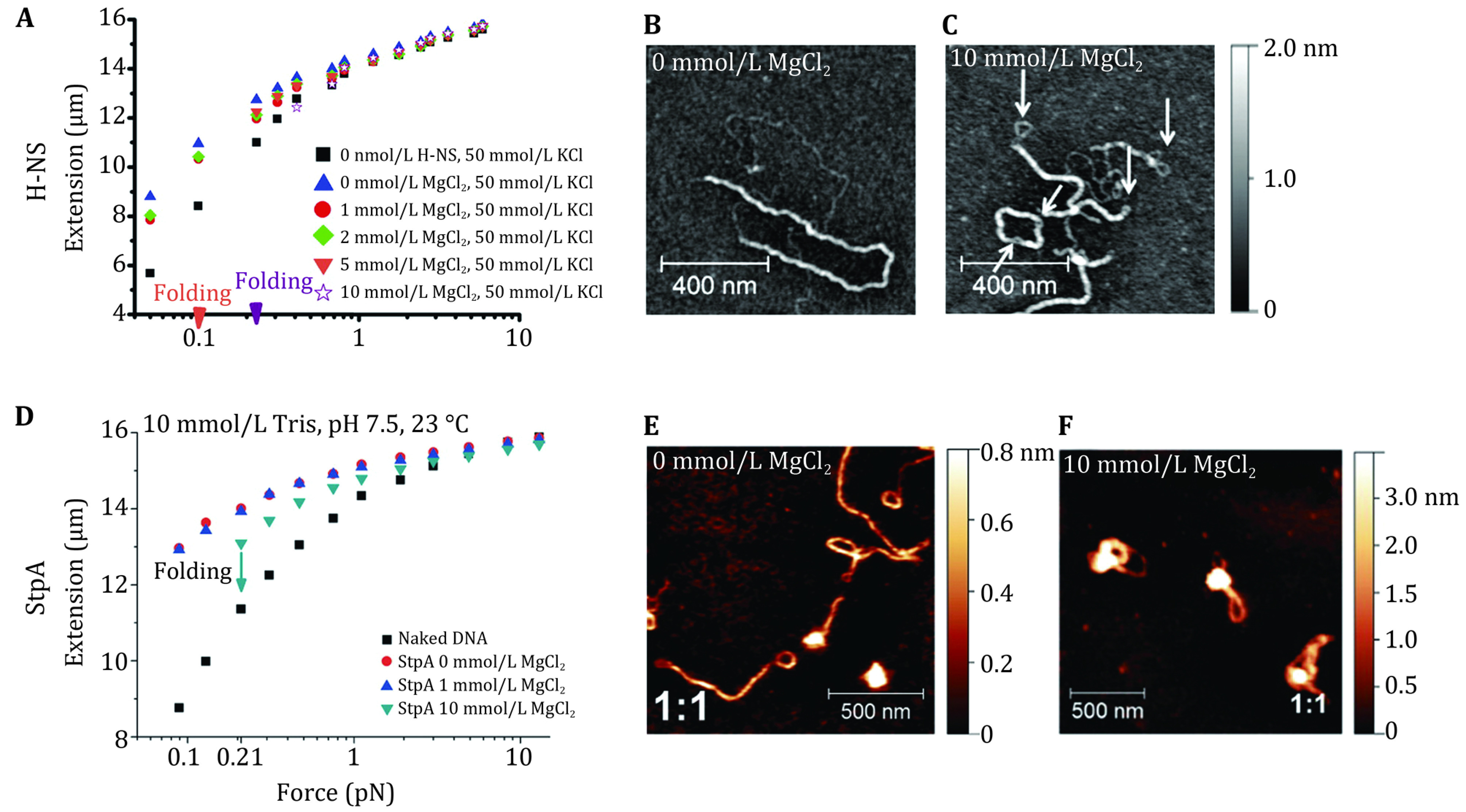
The single-DNA manipulation studies (**A**, **D**) and AFM imaging (**B**, **C**, **E**, **F**) of the nucleoprotein complexes formed by the H-NS family proteins. **A** The force-extension curve of a 49-kbp $ \lambda $-phage DNA in the presence of 600 nmol/L Salmonella H-NS at different solution conditions. The two arrows indicate the H-NS induced collapse of DNA extension. **B**, **C** AFM images of H-NS nucleoprotein filaments at different magnesium concentrations. **D** The force-extension curve (600 nmol/L StpA). **E**, **F** AFM images of the nucleoprotein complexes formed by the H-NS paralog, StpA, at different magnesium concentrations. Results show that continuous nucleoprotein filaments and high-order nucleoprotein complexes can be formed under different solution conditions

Recent studies of H-NS family proteins such as H-NS, StpA, MvaT, and MvaU from different bacterial species have reported that these proteins organize large DNA molecules into various conformations at different solution conditions. The AFM imaging showed that they form rigid continuous nucleoprotein filaments covering a large segment of DNA molecules (Amit* et al.* 2003; Lim* et al.* 2012b; Liu* et al.* 2010; Winardhi* et al.* 2012, 2014), large hairpin-like DNA structures, and higher-order DNA condensates (Dame* et al.* 2000; Lim* et al.* 2012b; Liu* et al.* 2010; Winardhi* et al.* 2012, 2014) (Fig. 5B, 5C, 5E and 5F) depending on solution pH and salt concentrations. Consistently, different levels of DNA extension or compaction by these H-NS family proteins at corresponding solution conditions were revealed by single-DNA manipulation experiments (Fig. 5A and 5D). An important finding from these studies is that magnesium is a crucial regulator of DNA conformation organized by these proteins. In general, when the concentration of magnesium increases over a physiological range of a few mmol/L, a switch from a simpler to a higher order nucleoprotein organization was observed (Lim* et al.* 2012b; Liu* et al.* 2010; Winardhi* et al.* 2012, 2014). In addition, some experiments have suggested a common binding mode of the H-NS family proteins, namely the locally spread extended formation of rigid protein filament with cooperative DNA binding to mediate the DNA conformations (Lim* et al.* 2012b; Winardhi* et al.* 2015). The protein filament further interacts with other DNA segments, leading to DNA bridging or the formation of higher order nucleoprotein complexes, in a manner dependent on the protein species and solution conditions.

#### IHF protein

The combined approach of AFM imaging and the single-DNA manipulation have also been utilized to investigate IHF, another bacterial NAP. IHF has a high intracellular concentration of 12–55 ${\text{μ}}$mol/L during the exponential growth phase and the early stationary phase of *E. coli* (Ali Azam* et al.* 1999). Single-DNA manipulation studies revealed that DNA bending is the primary binding mode of the non-specific DNA binding of IHF, with an averaged bending angle of ~$ 50° $ estimated at each binding site. AFM imaging showed that such a moderate level of DNA bending by IHF can significantly compact the conformations of large DNA molecules. Similar approaches have been applied to study other NAPs and their DNA-binding modes, such as HU bending and wrapping DNA (van Noort* et al.* 2004), and Dps bringing several remote DNA sites together via multiple DNA-binding sites on the protein forming highly compact nucleoprotein condensates (Ceci* et al.* 2004; Lee* et al.* 2015).

### Site-specific DNA binding by architectural proteins

#### H-NS protein

**Figure 6:**
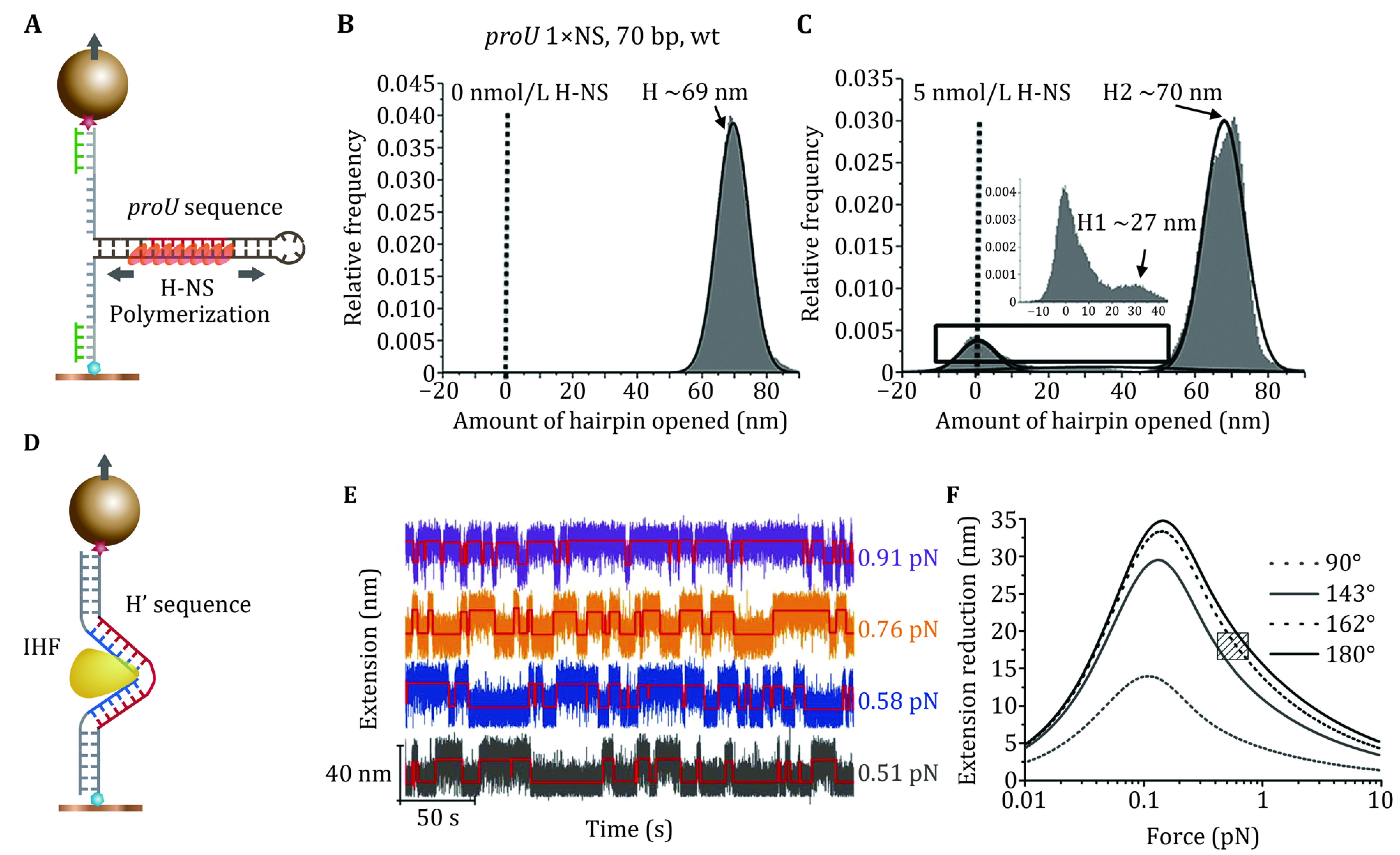
The single-DNA manipulation studies of site-specific protein binding to DNA. **A** H-NS binding to the *proU* sequence embedded in a dsDNA hairpin which is excluded from the force-transmission pathway. **B** In the absence of H-NS, the change in the extension of the DNA hairpin upon a hairpin-destabilizing force (~10 pN) indicates a fully unzipped state of the hairpin. **C** In the presence of 5 nmol/L H-NS, the change in the extension of the hairpin upon the same hairpin-destabilizing force indicates the spread of H-NS binding from the *proU* nucleation sequence that led to the stabilization of the entire hairpin (further details can be found in (Gulvady* et al.* 2018)). **D** IHF binding to an H' sequence embedded in a short dsDNA within the force-transmission pathway. **E** The association/dissociation of IHF to/from the H' sequence results in stepwise fluctuations between a lower dsDNA extension (H' bound) and a higher dsDNA extension (H' unbound). **F** Based on the force-dependent step sizes of the DNA extension fluctuation, the IHF binding induced DNA bending at the H' site was estimated to be in a range from 140 to 180 degrees (further details in Le* et al.* (2013))

Besides serving as NAPs to organize the bacterial nucleoid, H-NS family proteins are also known to regulate gene transcription mainly as gene silencers to target specific DNA sequence motifs (Bouffartigues* et al.* 2007; Lang* et al.* 2007) located at the promoter regions of the corresponding genes. H-NS nucleoprotein filaments are formed locally around the promotor sequences. That the nucleoprotein filaments polymerize from the nucleation site has been observed in a recent single-DNA footprinting assay. In this assay, mechanical manipulation was performed on a DNA hairpin containing a single nucleation site, which was excluded from the force transmission pathway (Fig. 6A–6C) (Gulvady* et al.* 2018). The dissociation constant of H-NS binding to the nucleation sequence from the *pro*U promotor was hence estimated to be around 10 nmol/L. Both the positively charged residues in the linker region and the C-terminal specific DNA binding domain in H-NS contribute to this DNA binding affinity. The continuous filaments formed by H-NS family proteins on the promotor regions could completely cover the promoter sequences. This covering of the promoter regions blocks the access of the transcription factors, thus, providing a natural mechanism for the gene-silencing function of the H-NS protein. Consistent with this view, a previous AFM study showed that H-NS mutants which cannot silence genes lost their abilities to form an extended H-NS nucleoprotein filament (Lim* et al.* 2012b). The gene silencing function of H-NS could be further enhanced by DNA bridging mediated by the H-NS nucleoprotein filament as proposed previously (Dame* et al.* 2000; Winardhi* et al.* 2015).

#### IHF protein

Like H-NS, IHF also has a highly sequence-specific function. IHF was originally discovered as an essential co-factor for site-specific recombination of phage l into *E. coli* genome (Goosen and van de Putte 1995; Lin 2013), which requires the binding of IHF to the H’ sequence (5'-TATCAA-3') (Lim* et al.* 2012a). The X-ray crystal structure showed that IHF creates a sharp (>$ 160° $) DNA kink at the H' binding site (Rice* et al.* 1996), facilitating the juxtaposition between two distal DNA-binding sites of the protein integrase necessary for the recombination reaction. The IHF induced DNA deformation at the H’ site was investigated by both AFM imaging and the single-DNA manipulation technologies (Le* et al.* 2013; Yoshua* et al.* 2021). A recent high-resolution AFM imaging suggested the existence of three topological modes of the IHF-H' complex with different bending angles, an “associated” mode (~73˚), a “half-wrapped” mode (~107˚) and a “fully-wrapped” mode (~147˚). The “fully-wrapped” mode was only observed at the H' site, whereas the other modes were also observed on DNA that does not contain the H' sequence. The bending angle of the “associated” mode is close to that of non-specific IHF binding to DNA, estimated based on the shift in the force-extension curves measured in a single-DNA manipulation experiment (Lim* et al.* 2012a). The bending angle of the fully wrapped mode is close to the value estimated based on the force-dependent stepwise fluctuation in the extension of a short DNA containing a single H' site in a recent single-DNA manipulation experiment (Fig. 6D–6F) (Le* et al.* 2013).

#### HMGA2 protein

**Figure 7:**
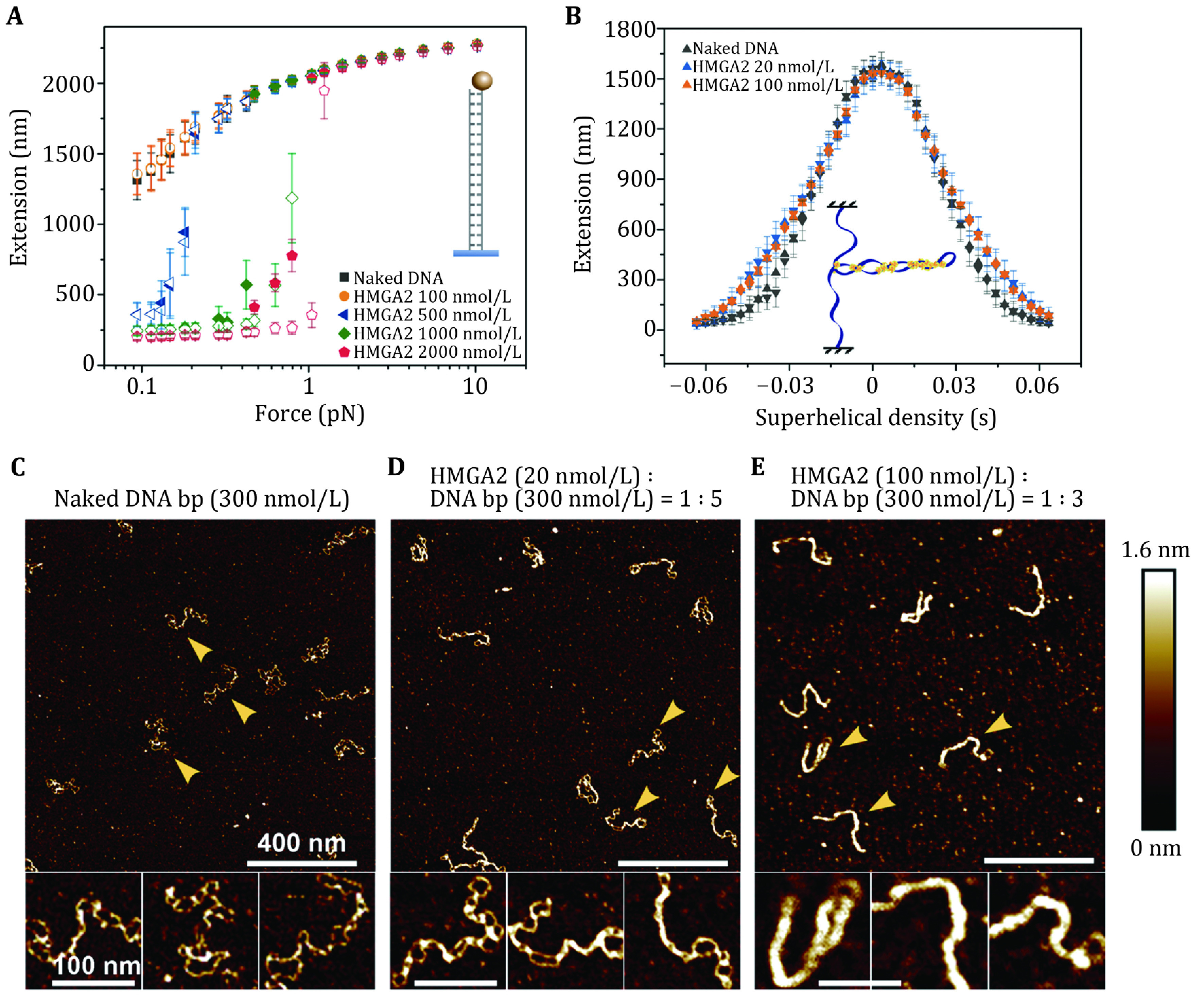
**A**, **B** The single-molecule manipulation of HMGA2 binding to a 6.5-kbp linking number-unconstrained dsDNA (**A**) and a linking number-constrained, supercoiled DNA (**B**) at different HMGA2 concentrations. **C**–**E** AFM images of a 2.7 kb negatively supercoiled DNA bound with HMGA2 at different HMGA2:DNA base pair stoichiometric ratios. We refer the readers to Zhao* et al.* (2017) for further details

The high mobility group (HMG) protein family is a group of nonhistone chromosomal proteins that are involved in the regulations of various DNA-dependent processes such as transcription, replication, recombination, and DNA repair (Bianchi and Agresti 2005; Bustin 1999; Ozturk* et al.* 2014; Reeves 2010). Recent studies have shown that HMGA2 preferentially recognizes DNA Holliday junctions and three-way junctions (Yu* et al.* 2014). The protein harbors three AT-hooks as the unique DNA-binding domains which preferentially recognize the minor groove of AT-rich duplex DNA sequences. The interaction between HMGA2 and DNA has been investigated by AFM imaging and the single-DNA manipulation approaches (Zhao* et al.* 2017, 2019). The single-DNA manipulation experiments for non-specific DNA sequences showed no shift in the force-extension curve of DNA from the bare DNA at protein concentrations below 100 nmol/L (Fig. 7A), indicating an insignificant binding of the protein in this protein concentration range. At higher protein concentrations, HMGA2 can mediate DNA condensation at sub-pN forces, suggesting DNA juxtaposition by HMGA2 occurs where more than one DNA site is bound on one HMGA2 (Fig. 7A).

Interestingly, on supercoiled DNA molecules, the binding of HMGA2 occurred at much lower protein concentrations (as low as 10 nmol/L), as revealed in both single-DNA manipulation (Fig. 7B) and AFM imaging (Fig. 7C–7E) studies (Zhao* et al.* 2017). The DNA crosses in the supercoiled plasmids bring distal DNA sites into physical proximity, which explains the higher binding affinity of HMGA2 to the supercoiled DNA conformation based on the decreased free energy cost for DNA juxtaposition. The DNA juxtaposition mechanism also explains the preferential binding of HMGA2 to DNA Holliday junctions and three-way junctions (Yu* et al.* 2014) where multiple DNA branches meet.

**Figure 8:**
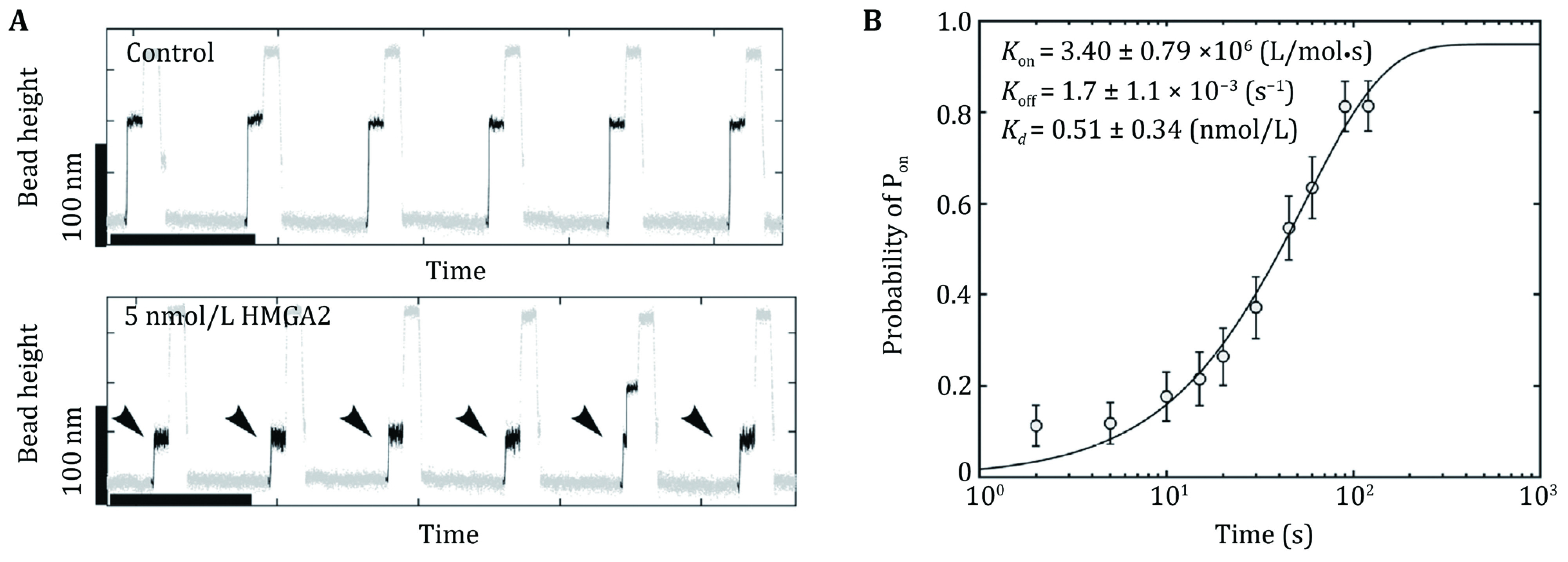
**A** HMGA2 binding to a specific sequence (5’-ATATTCGCGAWWATT-3’) embedded in a 52-bp dsDNA hairpin, which is excluded from the force transmission pathway. Representative time traces of the height change of a bead attached to the end of the DNA construct during cycles of sequential force jumps among 7.7 ± 0.8 (at the force the hairpin is stable), 12.1 ± 1.2 (at the force the naked hairpin is unstable) and 30.0 ± 3.0 pN (at the force the hairpin is unzipped and the bound HMGA2 is displaced), in the absence of HMGA2 (top panel) and with 5 nmol/L HMGA2 (bottom panel). **B** The probability $ p\left(\Delta t\right) $ of the specific site binding of an HMGA2 protein was obtained in a force-jump assay over a time window of $ \Delta t $ when the DNA construct was held at the force of ~7.7 pN, from which the association rate, dissociation rate and the dissociation constant were obtained (further details in Zhao* et al.* (2019))

Besides the preferential binding to certain DNA conformations and structures, HMGA2 also targets a high binding affinity sequence motif, 5'-ATATTCGCGAWWATT-3', with a dissociation constant in the nanomolar range. The association rate $ {k}_{\rm{o}\rm{n}} $ and dissociation rate $ {k}_{\rm{o}\rm{f}\rm{f}} $ of HMGA2 binding to the same consensus sequence were quantified by unzipping a DNA hairpin in a force-jump assay. The hairpin harbored a single protein binding site, which was excluded from the force transmission pathway (Fig. 3A). The binding of HMGA2 was detected based on the delay caused by the bound protein in unzipping the DNA hairpin (Zhao* et al.* 2019). The association rate, dissociation rate, and the dissociation constant were determined to be $ {k}_{\rm{o}\rm{n}}=3.40\pm 0.79\times {10}^{6}\;{\rm{L}/\rm{mol}}\cdot {\rm{s}} $, $ {k}_{\rm{o}\rm{f}\rm{f}}= 1.7\pm 1.1\times {10}^{-3}{\rm{s}}^{-1} $, and $ {K}_{d}=\dfrac{{k}_{\rm{o}\rm{f}\rm{f}}}{{k}_{\rm{o}\rm{n}}}=0.51\;\pm  $ $  0.34\;{\rm{nmol}}/{\rm{L}} $, respectively (Fig. 8A and 8B).

## SUMMARY AND PERSPECTIVES

In summary, we have provided an overview on the basics of AFM imaging and the single-DNA mechanical manipulation technologies, and their integrated applications using the studies of the architectural proteins on DNA binding. AFM imaging approach provides direct visual information on the global conformations of the DNA–protein complexes, whereas the single-DNA mechanical manipulation approach provides highly precise quantifications of the protein binding induced changes in the local DNA conformation, the kinetics, and the binding affinity of DNA–protein interactions. The reviewed examples demonstrate that the integration of these two highly complementary approaches is powerful in revealing the binding modes and quantifying the non-specific and site-specific DNA–protein interactions, thus, providing rich insights into the underlying mechanisms of architectural proteins functioning in the genome packaging, the regulations of DNA transcription, recombination, and the DNA repair.

## Conflict of interest

Xiaodan Zhao, Xuyao Priscilla Liu and Jie Yan declare that they have no conflict of interest.
